# Supplementary material for: TastepepAI: An artificial intelligence platform for taste peptide de novo design
Source: PLoS Comput Biol. 2025 Oct 16;21(10):e1013602. doi: 10.1371/journal.pcbi.1013602 (PMC12543283; doi:10.1371/journal.pcbi.1013602)
Supplement: S1 File — (ZIP) [file pcbi.1013602.s006.zip › Supplementary Data 2/TaPep58 HPLC.doc]

**HPLC REPORT**

| Sample: | NJP93362 TaPep58 LL-5 | | | Analyzed date: | 2024-9-26 |
| --- | --- | --- | --- | --- | --- |
| Analyst: | WJ | | | Reconstitution: | 100%H_2_O |
| Lot. No.: | NJP93362-1172285 | | | | |
| Column: | 4.6×250mm,Venusil MP C18-5 | | | | |
| Solvent A | A: 0.1% Trifluoroacetic Acid in 100% Acetonitrile | | | | |
| Solvent B | B: 0.1% Trifluoroacetic Acid in 100% Water | | | | |
| Gradient: |  | A | B |  | |
|  | 0.0min | 15% | 85% |  | |
|  | 25.0min | 40% | 60% |  | |
|  | 25.1min | 100% | 0% |  | |
|  | 30.0min | Stop | |  | |
| Volume: | 5μl | | |  | |
| Wavelength: | 220nm | | |  | |
| Flow rate: | 1.0ml/min | | |  | |

─────────────────────────────────────────────────────────────

Rank	Time	Conc. Area	Height

────────────────────────────────────────────────────────────

1	8.750	1.3916	150193	27691

2	8.899	96.9791	10466866	625805

3	9.450	0.2863	30901	4362

4	10.073	0.9644	104082	13092

5	10.815	0.3786	40862	5232

────────────────────────────────────────────────────────────

Total	100	10792904	676182
